# Impact of a mobile application (reminder app) on acute toxicity during radiotherapy of head-and-neck cancer – results of a randomized phase III trial (RAREST-02)

**DOI:** 10.1186/s12885-022-10088-3

**Published:** 2022-09-17

**Authors:** Dirk Rades, Inga Zwaan, Jon Cacicedo, Karl L. Bruchhage, Samer G. Hakim, Denise Olbrich, Steven E. Schild, Soeren Tvilsted, Stefan Janssen

**Affiliations:** 1 Department of Radiation Oncology, University of Lübeck, Ratzeburger Allee 160, 23562 Lübeck, Germany; 2 Department of Radiation Oncology, Cruces University Hospital/ Biocruces Bizkaia Health Research Institute, Barakaldo, Vizcaya Spain; 3 Department of Oto-Rhino-Laryngology and Head and Neck Surgery, University of Lübeck, Lübeck, Germany; 4 Department of Oral and Maxillofacial Surgery, University of Lübeck, Lübeck, Germany; 5 Centre for Clinical Trials Lübeck, Lübeck, Germany; 6 Department of Radiation Oncology, Mayo Clinic Scottsdale, Scottsdale, AZ USA; 7 Research Department, Zealand University Hospital, Køge, Denmark; 8 Medical Practice for Radiotherapy and Radiation Oncology, Hannover, Germany

**Keywords:** Mobile application, Acute toxicity, Radiotherapy, Radio-chemotherapy, Head-and-neck cancer

## Abstract

**Background:**

Radiotherapy of head-and-neck cancer (SCCHN) is often associated with acute toxicity. In a previous trial, daily reminders by staff members to perform skin care resulted in less dermatitis. This randomized trial investigated whether a mobile application can replace these reminders.

**Methods:**

Patients were stratified according to tumor site, treatment and center. Fifty-three patients were eligible for per-protocol-set (25 with, 28 without app). Primary endpoint was grade ≥ 2 dermatitis until 60 Gy. Secondary endpoints included dermatitis grade ≥ 2 until end of radiotherapy (EOT), dermatitis grade ≥ 3, and mucositis grade ≥ 2 and ≥ 3.

**Results:**

After an interim analysis, the study was terminated (delayed and slow accrual). Until 60 Gy, grade ≥ 2 dermatitis rates were 72% with vs. 82% without app (*p* = 0.38), grade ≥ 3 dermatitis rates 20% vs. 11% (*p* = 0.45). Until EOT, grade ≥ 2 and ≥ 3 dermatitis rates were 72% vs. 86% (*p* = 0.22) and 24% vs. 18% (*p* = 0.58). Until 60 Gy, grade ≥ 2 and ≥ 3 mucositis rates were 76% vs. 82% (*p* = 0.58) and 20% vs. 36% (*p* = 0.20). Until EOT, corresponding mucositis rates were 76% vs. 82% (*p* = 0.58) and 28% vs. 43% (*p* = 0.26).

**Conclusion:**

Given the limitations of this trial, the reminder app led to non-significant reduction of grade ≥ 2 dermatitis, grade ≥ 2 mucositis and ≥ 3 mucositis. Additional studies are required to define the value of reminder apps during radiotherapy for SCCHN.

## Background

Radiotherapy and radio-chemotherapy are frequently used for treating locally advanced squamous cell head-and-neck cancer (SCCHN) [1]. This treatment is often associated with acute toxicity including dermatitis and oral mucositis, particularly if simultaneous radio-chemotherapy is administered. Severe (grade ≥ 3) toxicity may require interruption of the radiation treatment, which can have a negative effect on the treatment results, particularly on loco-regional control [2, 3]. Therefore, grade 3 toxicities should be avoided. To achieve this goal, it is important to postpone lower grade toxicities as long as possible or avoid them completely.

A previous randomized phase III trial tested a new wound dressing and compared it to standard skin care with creams and lotions [4, 5]. It was assumed that the dressing would reduce the rate of grade ≥ 2 dermatitis until 50 Gy from 85 to 65%. Surprisingly, the grade ≥ 2 dermatitis rates were significantly lower (less than 40%) in both groups [5]. It was hypothesized that daily reminders by staff members to perform skin care, which were part of the study protocol, improved the patients’ compliance and consecutively led to a decrease of the dermatitis rate in the control group. If this theory was correct, one may further speculate whether the reminders by the staff members can be replaced by reminders from a mobile application (reminder app).

Therefore, the current randomized phase III trial (RAREST-02) was performed that compared standard skin and mouth care supported by a reminder app to standard care alone in patients irradiated for locally advanced SCCHN with respect to radiation-induced dermatitis and oral mucositis. It was assumed that using the reminder app would decrease the rates of grade ≥ 2 dermatitis and mucositis occurring until 60 Gy by 20%.

## Methods

This randomized phase III trial (active-controlled, parallel-group) compared standard skin and mouth care supported by a reminder app (experimental arm) to standard care alone (control arm) with respect to acute toxicity in terms of dermatitis and oral mucositis in patients irradiated for SCCHN. The trial was approved by ethics committees responsible for the three contributing centers in Lübeck, Hannover and Barakaldo (initial approval by the ethics committee at the University of Lübeck, reference number 19–302), registered at clinicaltrials.gov (identifier: NCT04110977; URL: https://clinicaltrials.gov/show/NCT04110977) on 01/10/2019, and performed in accordance with the Declaration of Helsinki. To be included, patients were required to have histologically proven SCCHN and to be able to use a smartphone. The complete inclusion and exclusion criteria were described in a previous article [6]. After randomization, patients were excluded from the per-protocol-set and the analyses, if they received less than 60 Gy and the reason for discontinuation of radiotherapy was not death, dermatitis or oral mucositis. They were also excluded if they had an interruption of radiotherapy for more than 7 days, if the reason was not dermatitis or oral mucositis. Patients who refused to use the reminder app or experienced problems with its download after having given informed consent to participate in the trial were included in the per-protocol-set (“crossover patients”) and analyzed as part of the control group.

Sixty patients were randomized (32 with app, 28 without app) between August 2020 and November 2021. Twenty-four patients could not be included in the trial, because they possessed only a mobile phone without smartphone features (*n* = 14) or no mobile phone at all (*n* = 10). Stratification was performed according to tumor site (oropharynx/oral cavity vs. hypopharynx/larynx), type of treatment (radio-chemotherapy vs. radiotherapy alone) and participating center (Lübeck vs. Hannover vs. Barakaldo). A stratified block-randomization with random block size was performed. The randomization was performed software-based at the Institute of Medical Biometry and Statistics Lübeck. A list including centrally prepared envelope inlays was given to an independent person at the coordinating investigators site. The list was distributed in pdf-format via a password-protected computed disc using a sealed envelope. The inlays were printed and put into the envelopes by a person not involved in the trial who signed a confidentiality agreement. The randomization envelopes were placed at the participating centers prior to the start of recruitment. The proceeding for randomization was based on standard operating procedures of the Institute of Medical Biometry and Statistics. Once the randomization was allocated to a patient it could not be changed anymore.

At the time of the interim analysis, 56 patients had completed curative radiotherapy. However, two patients receiving 66 Gy who had interruptions of their radiotherapy of 8 days and 12 days, respectively, and one patient receiving 66.6 Gy in 37 fractions of 1.8 Gy were excluded from further analyses. Thus, 53 patients qualified for the per-protocol-set (25 with app, 28 without app including 4 crossover patients). The characteristics of these patients in the experimental and the control arm are summarized in Table 1.

**Table 1:** Distribution of baseline characteristics in the experimental arm and the control arm

	**Experimental arm (with reminder app) N patients (%)**	**Control arm (without reminder app) N patients (%)**	***p*-value**
**Type of treatment**			0.98^a^
Radiotherapy alone	9 (36)	10 (36)	
Radio-chemotherapy	16 (64)	18 (64)	
**Radiotherapy dose**			0.77^a^
60 Gy	9 (36)	9 (32)	
64–70 Gy	16 (64)	19 (68)	
**Type of chemotherapy**			0.90^a^
Cisplatin, 2 cycles of 5 × 20 or 4 × 25 mg/m^2^	6 (38)	9 (50)	
Cisplatin, 2–3 cycles of 1 × 100 mg/m^2^	3 (19)	3 (17)	
Cisplatin, 40 mg/m^2^ weekly	6 (38)	5 (28)	
Carboplatin (4 × AUC 1.5 / 5 × AUC 1.0)	1 (6)	1 (6)	
**Tumor site**			0.80^a^
Hypopharynx	3 (12)	2 (7)	
Larynx	4 (16)	3 (11)	
Oral cavity	5 (20)	8 (29)	
Oropharynx	13 (52)	15 (54)	
**Tumor site (combined)**			0.51^b^
Hypopharynx, larynx	7 (28)	5 (18)	
Oropharynx, oral cavity	18 (72)	23 (82)	
**Gender**			0.16^b^
Female	7 (28)	3 (11)	
Male	18 (72)	25 (89)	
**Age at start of radiotherapy**			0.27^a^
≤ 59 years	15 (60)	12 (43)	
≥ 60 years	10 (40)	16 (57)	
**ECOG performance status**			0.56^a^
0	16 (64)	20 (71)	
≥ 1	9 (36)	8 (29)	
**Primary tumor stage**			0.75^a^
T1-2	10 (40)	10 (36)	
T3-4	15 (60)	18 (64)	
**Nodal stage**			0.93^a^
N0-1	14 (56)	16 (57)	
N2-3	11 (44)	12 (43)	
**HPV-status**			0.40^a^
Negative	8 (32)	9 (32)	
Positive	11 (33)	16 (57)	
Unknown	6 (34)	3 (11)	
**Upfront surgery**			0.82^a^
No	6 (24)	6 (21)	
Yes	19 (76)	22 (79)	
**Neck Dissection**			0.96^a^
Bilateral	9 (36)	9 (32)	
Unilateral	10 (40)	12 (43)	
No	6 (24)	7 (25)	

^a^Chi-square test

^b^Fisher’s exact test

In the 53 patients included in the per-protocol-set, radiotherapy was performed as volumetric modulated arc therapy (VMAT) with conventional fractionation (2.0 Gy per fraction on five consecutive days per week). Further details of radiotherapy were previously reported [6]. Forty-one patients received upfront resection of the primary tumor plus dissection of loco-regional lymph nodes. Of these patients, 18 received radiotherapy alone (17 × 60 Gy, 1 × 66 Gy) and 23 patients with risk factors (2 × incomplete resection, 18 × extra-capsular extension of lymph nodes, 3 × both) received concurrent radio-chemotherapy (1 × 60 Gy, 1 × 64 Gy, 19 × 66 Gy, 2 × 70 Gy). These patients received cisplatin with two cycles of 5 × 20 mg/m^2^ or 4 × 25 mg/m^2^ (*n* = 12), weekly administration of 40 mg/m^2^ (*n* = 8), or two to three cycles of 1 × 100 mg/m^2^ (*n* = 2). One patient scheduled for 5 × 20 mg/m^2^ received two cycles of carboplatin (4 × AUC 1.5 and 5 × AUC 1.0, respectively). Cumulative cisplatin doses were ≥ 200 mg/m^2^ in 16 patients and < 200 mg/m^2^ in 7 patients. Twelve patients received definitive treatment with 70 Gy of radiotherapy, either alone (*n* = 1) or combined with concurrent chemotherapy (*n* = 11). Chemotherapy regimens included cisplatin with two cycles of 5 × 20 mg/m^2^ or 4 × 25 mg/m^2^ (*n* = 3), weekly administration of 40 mg/m^2^ (*n* = 3) or two to three cycles of 1 × 100 mg/m^2^ (*n* = 4). One patient scheduled for 5 × 20 mg/m^2^ received two cycles of carboplatin (4 × AUC 1.5 and 5 × AUC 1.0, respectively). Cumulative cisplatin doses were ≥ 200 mg/m^2^ in 9 and < 200 mg/m^2^ in 2 patients.

### Standard care and reminder app

In both the experimental and the control arm, standard skin and mouth care were performed by patients from the start of radiotherapy, and all patients received corresponding paper-based instructions prior to the start of their treatment. Skin care included fatty cream (with or without urea) and mometasone furoate cream. It was continued up to one week following EOT or until moist desquamation or grade ≥ 3 dermatitis occurred. Moist desquamation or grade ≥ 3 dermatitis was treated with antiseptic agents followed by silicon or calcium alginate bandage. Mouth care was performed with antibacterial mouth rinses (4 times per day), supplemented by lidocaine hydrochloride plus dexpanthenole solution, benzdyamine hydrochloride solution or even systemic analgesics in case of pain. Oral edema was treated with hydrocortisone acetate if necessary. Patients of the experimental arm were supported by the reminder app “CareReminder”, which was developed specifically for this trial by Nextlabel OHG, Lübeck. The app reminded the patients four times a day to perform their skin and mouth care. Patients were seen by a radiation oncologist once a week and asked whether the app was working well. However, it was not regularly checked whether the app was disabled (intentionally or not) by the patients.

### Endpoints, statistical considerations, and interim analysis

Primary endpoint was grade ≥ 2 radiation dermatitis until 60 Gy. Dermatitis according to CTCAE v4.03 [7] was recorded weekly by two observers until EOT. In case of inconsistent graduation, a third observer was consulted. Secondary endpoints included radiation dermatitis grade ≥ 2 until end of radiotherapy (EOT), radiation dermatitis grade ≥ 3 until 60 Gy and EOT, oral mucositis grade ≥ 2 until 60 Gy and EOT, and oral mucositis ≥ 3 until 60 Gy and EOT. Oral mucositis according to the Radiation Therapy Oncology Group (RTOG) criteria [8–10] was also assessed weekly by two (or three) observers weekly until EOT. In previous studies, grade ≥ 2 dermatitis and mucositis rates were 86–92% and 86–100%, respectively [11–13]. Based on these data, a grade ≥ 2 radiation dermatitis rate of 85% was assumed for the control arm (“worst-case” scenario). The impact of the reminder app was considered clinically significant in case of a decrease of this rate by 20%. According to sample size calculations, 168 patients should be randomized including 5% of patients not qualifying for analyses to achieve a statistical power of 80%. Details of these calculations were previously reported [6]. The experimental and the control group were compared with respect to distributions of baseline characteristics and the rates of radiation dermatitis and oral mucositis using the Chi-square test or the Fisher’s exact test, if the expected frequency in a two-by-two table was < 5 in at least one cell. *P*-values < 0.05 were considered significant.

After completion of the radiotherapy in one third of the required number of patients, a planned interim analysis was performed, and it was discussed whether the trial would be continued and subgroup analyses would be reasonable. Of 56 patients who had completed curative radiotherapy at the time of the interim analysis, 53 patients qualified for the per-protocol-set. Since the accrual was much slower than expected, it was decided to stop the trial at this stage. Moreover, it was felt that it was reasonable to perform subgroup analyses (grade ≥ 2 and ≥ 3 toxicities until 60 Gy) for the clinical factors used for stratification, namely for SCCHN of oropharynx or oral cavity, SCCHN of hypopharynx or larynx, radio-chemotherapy, and radiotherapy alone. Due to the premature termination of the trial and the significantly reduced sample size, statistical analyses were descriptive in nature and focused on the per-protocol-set only.

## Results

After the planned interim analysis, the study was terminated. The start of the trial (first patient in) was delayed from October 2019 until August 2020 due to data protection issues (including a contract between the sponsor and Nextlabel OHG) and the Covid-19 pandemic. Moreover, accrual was slower than expected (60 patients in 15 months instead of 84 patients in 12 months), since a considerable number of patients screened for eligibility had no smartphone, did not wish to participate in the trial, or were already included in another clinical trial. Until 60 Gy, grade ≥ 2 radiation dermatitis occurred in 72% (18/25) of patients supported by the reminder app and 82% (23/28) of patients receiving standard care alone (*p* = 0.38) (Table 2). Grade ≥ 3 dermatitis rates until 60 Gy were 20% (5/25) and 11% (3/28), respectively (*p* = 0.45). Until EOT, grade ≥ 2 dermatitis rates were 72% (18/25) and 86% (24/28), respectively (*p* = 0.22), and grade ≥ 3 dermatitis rates were 24% (6/25) and 18% (5/28), respectively (*p* = 0.58).

**Table 2:** Comparison of the experimental arm and the control arm with respect to radiation dermatitis

	**Experimental arm (with reminder app) N patients (%)**	**Control arm (without reminder app) N patients (%)**	***p*-value**
**Dermatitis (worst grade) until 60 Gy**			0.86^a^
Grade 1	7 (28)	5 (18)	
Grade 2	13 (52)	20 (71)	
Grade 3	5 (20)	3 (11)	
**Dermatitis grade ≥ 2 until 60 Gy**	18 (72)	23 (82)	0.38^b^
**Dermatitis grade ≥ 3 until 60 Gy**	5 (20)	3 (11)	0.45^c^
**Dermatitis (worst grade) until EOT**			0.83^a^
Grade 1	7 (28)	4 (14)	
Grade 2	12 (48)	19 (68)	
Grade 3	6 (24)	5 (18)	
**Dermatitis grade ≥ 2 until EOT**	18 (72)	24 (86)	0.22^b^
**Dermatitis grade ≥ 3 until EOT**	6 (24)	5 (18)	0.58^b^

^a^Exact Wilcoxon Mann–Whitney two-sample test

^b^Chi-square test

^c^Fisher’s exact test

Until 60 Gy, grade ≥ 2 oral mucositis occurred in 76% (19/25) of patients in the experimental arm and 82% (23/38) of patients on the control arm (*p* = 0.58) (Table 3). Grade ≥ 3 mucositis rates until 60 Gy were 20% (5/25) and 36% (10/28), respectively (*p* = 0.20). Until EOT, grade ≥ 2 mucositis rates were 76% (19/25) and 82% (23/28), respectively (*p* = 0.58), and ≥ 3 mucositis rates were 28% (7/25) and 43% (12/28), respectively (*p* = 0.26). In the subgroup analyses, no significant differences regarding radiation dermatitis and oral mucositis were found. In the subgroup analysis of patients with cancer of the oropharynx or oral cavity, trends (*p* ≤ 0.20) were found for reduction of grade ≥ 2 and ≥ 3 mucositis until 60 Gy (reduction by 18% and 26%, respectively). Moreover, in patients receiving radiotherapy alone, a trend was found for reduction of grade ≥ 3 mucositis until 60 Gy (reduction by 39%), and in patients receiving radio-chemotherapy a trend for reduction of grade ≥ 2 dermatitis until 60 Gy (reduction by 19%). The results of the subgroup analyses are summarized in Tables 4, 5, 6 and 7.

**Table 3:** Comparison of the experimental arm and the control arm with respect to oral mucositis

	**Experimental arm (with reminder app) N patients (%)**	**Control arm (without reminder app) N patients (%)**	***p*-value**
**Mucositis (worst grade) until 60 Gy**			0.31^a^
Grade 0	0 (0)	1 (4)	
Grade 1	6 (24)	4 (14)	
Grade 2	14 (56)	13 (46)	
Grade 3	5 (20)	10 (36)	
**Mucositis grade ≥ 2 until 60 Gy**	19 (76)	23 (82)	0.58^b^
**Mucositis grade ≥ 3 until 60 Gy**	5 (20)	10 (36)	0.20^b^
**Mucositis (worst grade) until EOT**			0.34^a^
Grade 0	0 (0)	1 (4)	
Grade 1	6 (24)	4 (14)	
Grade 2	12 (48)	11 (39)	
Grade 3	7 (28)	12 (43)	
**Mucositis grade ≥ 2 until EOT**	19 (76)	23 (82)	0.58^b^
**Mucositis grade ≥ 3 until EOT**	7 (28)	12 (43)	0.26^b^

^a^Exact Wilcoxon Mann–Whitney two-sample test

^b^Chi-square test

**Table 4:** Subgroup analysis of patients with cancer of the oropharynx or oral cavity with respect to radiation dermatitis and oral mucositis

	**Experimental arm (with reminder app) N patients (%)**	**Control arm (without reminder app) N patients (%)**	***p*-value**^a^
**Dermatitis grade ≥ 2 until 60 Gy**	13 (72)	20 (87)	0.27
**Dermatitis grade ≥ 3 until 60 Gy**	3 (17)	2 (9)	0.64
**Mucositis grade ≥ 2 until 60 Gy**	14 (78)	22 (96)	0.15
**Mucositis grade ≥ 3 until 60 Gy**	3 (17)	10 (43)	0.096

^a^Fisher’s exact test

**Table 5:** Subgroup analysis of patients with cancer of the hypopharynx or larynx with respect to radiation dermatitis and oral mucositis

	**Experimental arm (with reminder app) N patients (%)**	**Control arm (without reminder app) N patients (%)**	***p*-value**^a^
**Dermatitis grade ≥ 2 until 60 Gy**	5 (71)	3 (60)	1.00
**Dermatitis grade ≥ 3 until 60 Gy**	2 (29)	1 (20)	1.00
**Mucositis grade ≥ 2 until 60 Gy**	5 (71)	1 (20)	0.24
**Mucositis grade ≥ 3 until 60 Gy**	2 (29)	0 (0)	0.47

^a^Fisher’s exact test

**Table 6:** Subgroup analysis of patients treated with radiotherapy alone with respect to radiation dermatitis and oral mucositis

	**Experimental arm (with reminder app) N patients (%)**	**Control arm (without reminder app) N patients (%)**	***p*-value**^a^
**Dermatitis grade ≥ 2 until 60 Gy**	6 (67)	6 (60)	1.00
**Dermatitis grade ≥ 3 until 60 Gy**	0 (0)	2 (20)	0.47
**Mucositis grade ≥ 2 until 60 Gy**	8 (89)	8 (80)	1.00
**Mucositis grade ≥ 3 until 60 Gy**	1 (11)	5 (50)	0.14

^a^Fisher’s exact test

**Table 7:** Subgroup analysis of patients treated with radio-chemotherapy with respect to radiation dermatitis and oral mucositis

	**Experimental arm (with reminder app) N patients (%)**	**Control arm (without reminder app) N patients (%)**	***p*-value**^a^
**Dermatitis grade ≥ 2 until 60 Gy**	12 (75)	17 (94)	0.16
**Dermatitis grade ≥ 3 until 60 Gy**	5 (31)	1 (6)	0.08
**Mucositis grade ≥ 2 until 60 Gy**	11 (69)	15 (83)	0.43
**Mucositis grade ≥ 3 until 60 Gy**	4 (25)	5 (28)	1.00

^a^Fisher’s exact test

## Discussion

Radiotherapy of SCHNN can be associated with significant toxicity including dermatitis and oral mucositis, particularly if it is combined with simultaneous chemotherapy. In the randomized trial of Calais et al. from 1999 that compared definitive radio-chemotherapy to radiotherapy alone for advanced-stage cancer of the oropharynx, 67% and 59% of the patients, respectively, experienced grade ≥ 3 dermatitis, and 71% and 39% of the patients, respectively, grade ≥ 3 mucositis [14]. Dermatitis and mucositis rates may also be high when modern radiotherapy techniques such as intensity-modulated radiation therapy (IMRT) are used. For example, in the prospective observational study of Kucha et al*.* that compared three-dimensional conformal radiotherapy (3D-CRT) and IMRT, grade ≥ 2 dermatitis and mucositis rates were 90% and 100%, respectively, in the IMRT-group [15]. Grade ≥ 3 dermatitis and mucositis rates were 5% and 21%, respectively. In the randomized prospective study of Grover et al*.*, patients receiving IMRT with a sequential boost had grade ≥ 2 dermatitis and mucositis rates of 59% and 88%, respectively, and corresponding grade ≥ 3 rates of 45% and 14%, respectively [16]. In another randomized study, patients receiving IMRT plus weekly cisplatin grade ≥ 2 dermatitis and mucositis rates were 72% and 100%, respectively, and corresponding grade ≥ 3 rates were 12% and 60%, respectively [17].

Severe acute toxicity may lead to interruption of radiotherapy, which was reported to impair the outcomes of radiotherapy or radio-chemotherapy. In the multivariate analysis of a retrospective study from 2008, patients without interruptions of radiotherapy for longer than one week had significantly better loco-regional control (risk ratio 3.32, 95% confidence interval: 1.26 – 8.79, *p* = 0.015) and survival (risk ratio: 2.59, 95% confidence interval: 1.15 – 5.78, *p* = 0.021) [2]. Another study investigated interruptions of radiotherapy in elderly (≥ 66 years) Medicare beneficiaries with head-and-neck cancers identified from a surveillance, epidemiology, and end results-Medicare linked database [3]. Patients with larynx cancer and interruption of their treatment had a significantly increased risk of death by 68% (95% confidence interval: 41%-200%) compared to patients without an interruption. Similar associations were found for other tumor sites but differences did not achieve statistical significance, most likely due to small sample sizes. Moreover, in the review article of Ferreira et al*.*, a strong relationship was found between prolongation of the overall treatment time and loco-regional control and/or survival in patients irradiated for head-and-neck cancers [18]. It was concluded that such a prolongation may result in an average decrease of loco-regional control ranging from 1.2% per day to 12–14% per week.

These data illustrate that interruptions of treatment due to acute toxicity should be avoided in patients irradiated for SCCHN. It is important to decrease grade 2 adverse events or at least postpone their occurrence so they don’t get worse (grade ≥ 3). In the previous studies used for the sample size calculations of the present trial, 86–92% of patients receiving radiotherapy or radio-chemotherapy for SCCHN experienced grade ≥ 2 radiation dermatitis and 86–100% grade ≥ 2 oral mucositis, respectively [1, 11–13]. These unacceptably high toxicity rates were found despite standard skin and mouth care. One possible explanation is the limited compliance of the patients, since daily skin and mouth care, which needs to be performed several times per day, requires a high level of discipline. This hypothesis was supported by the results of a previous randomized phase III trial that compared a new absorbent, self-adhesive dressing (experimental arm) to standard skin care (control arm) with respect to prevention of radiation dermatitis [4, 5]. In this trial, the dermatitis rates were significantly lower than expected in both arms, i.e. also in the control arm. This unexpected result was believed to be due to the fact that the patients received daily reminders by at least two medical staff members (instead of routinely once a week by one staff member) to perform their skin care. This likely led to a better compliance, more intensive and regular skin care, and consecutively a reduction in radiation-induced dermatitis. Therefore, besides novel agents for skin and mouth care, new approaches are required that can improve the patients’ compliance.

However, due to limited personal resources, it is difficult to remind the patients every day, particularly in times of high patient load. Therefore, alternative options are required. One option could be a mobile application that reminds the patients every day to perform their skin and mouth care. The present randomized trial investigated the effect of such a reminder app on radiation dermatitis and oral mucositis by comparing standard care supported by an app to standard care alone. According to the sample size calculations, a total of 168 patients (including 5% not qualifying for the analyses) were required. Interim analyses were planned after completion of radiotherapy in one third (*n* = 56) and two thirds (*n* = 112) of the patients. At the time of the first interim analysis, it was decided to prematurely terminate the trial, mainly due to delayed and slow accrual of patients. In addition to its early termination, the RAREST-02 trial had further limitations. Only 53 of the 56 patients who had completed curative radiotherapy, did qualify for the analyses within the per-protocol-set. Although the distribution of chemotherapy types was not significantly different between the experimental arm and the control arm, an impact of the type of chemotherapy on the study results, particularly regarding oral mucositis, could not be completely excluded. Moreover, the fact that patients without a smartphone were not eligible for participating in the trial, has led to a selection bias. Patients without a smartphone may be older, less interested in technology, and of lower social status when compared to patients possessing a smartphone. This problem could have been solved by including patients with mobile phones without smartphone features and sending them SMS reminders. However, due to data protection regulations, SMS reminders were not allowed in this trial. After discussions with the corresponding authorities, only the use of a reminder app was possible, and the patient’s e-mail address had to be deleted by Nextlabel OHG immediately after the download of the app. No further transfer of patient-related data was required. To send regular SMS reminders, the patient’s telephone number must have been stored. The fact that it was not regularly checked whether patients disabled the app, was another major methodological drawback of this study. Because of these limitations, the results reported here should be interpreted with caution. According to these results, the use of the reminder app in addition to standard skin and mouth care was associated with non-significantly less grade ≥ 2 dermatitis, grade ≥ 2 mucositis and grade ≥ 3 mucositis. No reduction was found for grade ≥ 3 dermatitis until 60 Gy and EOT. This may be explained by the small numbers of events (8 and 11, respectively) regarding this endpoint, which were lower than for the other endpoints. In the subgroup analyses, several trends were found for reduction of dermatitis and mucositis until 60 Gy; rates of reduction ranged between 18 and 39%. However, the number of patients in these subgroup analyses appeared too small to draw valid conclusions. One may speculate whether some of the observed differences would have achieved statistical significance, if the trial was completed regularly.

In summary, the reminder app led to non-significant reduction of grade ≥ 2 dermatitis and grade ≥ 2 and ≥ 3 mucositis. The limitations of this trial, mainly its early termination, need to be considered when interpreting the results. Additional randomized trials are required to properly define the value of a reminder app to reduce the acute toxicity during radiotherapy of SCCHN.

## Data Availability

This trial was registered at clinicaltrials.gov (identifier: NCT04110977), where further details are available. For requests regarding data from this study, please contact the corresponding author (dirk.rades@uksh.de).

## Abbreviations

CTCAE: Common Terminology Criteria for Adverse Events
ECOG: Eastern Cooperative Oncology Group
EOT: End of radiation treatment
HPV: Human papilloma virus
OHG: Offene Handelsgesellschaft
RAREST: Radiotherapy related skin toxicity
RTOG: Radiation Therapy Oncology Group
SCCHN: Squamous Cell Carcinoma of the head-and-neck
VMAT: Volumetric Modulated Arc therapy
